# The Seasonality of Nitrite Concentrations in a Chloraminated Drinking Water Distribution System

**DOI:** 10.3390/ijerph15081756

**Published:** 2018-08-15

**Authors:** Pirjo-Liisa Rantanen, Ilkka Mellin, Minna M. Keinänen-Toivola, Merja Ahonen, Riku Vahala

**Affiliations:** 1 Department of Built Environment, School of Engineering, Aalto University, 02150 Espoo, Finland; riku.vahala@aalto.fi; 2 Department of Mathematics and Systems Analysis, Aalto University, School of Science, 02150 Espoo, Finland; ilkka.mellin@aalto.fi; 3 Faculty of Technology, Satakunta University of Applied Sciences, 26101 Rauma, Finland; minna.keinanen-toivola@samk.fi (M.M.K.-T.); merja.ahonen@samk.fi (M.A.)

**Keywords:** nitrite, disinfection by-product, drinking water distribution systems, seasonality

## Abstract

We studied the seasonal variation of nitrite exposure in a drinking water distribution system (DWDS) with monochloramine disinfection in the Helsinki Metropolitan Area. In Finland, tap water is the main source of drinking water, and thus the nitrite in tap water increases nitrite exposure. Our data included both the obligatory monitoring and a sampling campaign data from a sampling campaign. Seasonality was evaluated by comparing a nitrite time series to temperature and by calculating the seasonal indices of the nitrite time series. The main drivers of nitrite seasonality were the temperature and the water age. We observed that with low water ages (median: 6.7 h) the highest nitrite exposure occurred during the summer months, and with higher water ages (median: 31 h) during the winter months. With the highest water age (190 h), nitrite concentrations were the lowest. At a low temperature, the high nitrite concentrations in the winter were caused by the decelerated ammonium oxidation. The dominant reaction at low water ages was ammonium oxidation into nitrite and, at high water ages, it was nitrite oxidation into nitrate. These results help to direct monitoring appropriately to gain exact knowledge of nitrite exposure. Also, possible future process changes and additional disinfection measures can be designed appropriately to minimize extra nitrite exposure.

## 1. Introduction

Disinfection of drinking water is necessary to maintain hygienically good water quality in large drinking water distribution systems (DWDSs). When monochloramine (NH_2_Cl) is used for secondary disinfection in DWDSs, nitrite is quite often formed as a disinfection by-product (DBP). Nitrite is a potentially harmful compound when ingested by humans. In humans, nitrite can cause methemoglobinemia, which is a specific type of anemia. Infants younger than one year are more susceptible to methemoglobinemia than older children and adults. In methemoglobinemia, nitrite reacts with the hemoglobin of blood and disrupts oxygen transfer [1]. Furthermore, nitrite has been observed to cause several types of cancer in animals and may potentially do so in humans. Moreover, nitrite has been found to be potentially cancerous in animal tests, when ingested together with amines and amides or in conditions where the formation of organic nitroso compounds is possible [2].

Monochloramine is often favored in secondary disinfection because it significantly reduces the formation of trihalomethanes and other chlorination by-products compared to other forms of chlorine [3]. Nevertheless, when monochloramine decomposes in disinfection or during other reactions, ammonium is formed. Frequently, ammonium reacts to form nitrite in a biochemical oxidation reaction, called nitrification, in the biofilms on the inner surface of the pipes and reservoirs of the DWDS. In such cases, typical nitrite concentrations are 0.05–0.5 mgN l^−1^, but concentrations above 1 mgN l^−1^ have been observed in the stagnant parts of some DWDSs [4,5]. Furthermore, nitrite concentrations in drinking water can originate from polluted groundwater [6]. Due to the potential toxicity of nitrite, its concentrations are regulated in drinking water. In Finland, the statutory limit for nitrite in drinking water is 0.5 mg l^−1^ (0.15 mgN l^−1^) [7], following EU legislation [8]. The statutory limits require constant monitoring of the nitrite concentrations in distributed drinking water. 

This article discusses the seasonal patterns of nitrite concentrations in drinking water. A term often connected to monochloramine use in DWDSs is a nitrification episode, which in itself implies that nitrification and nitrite occur in relation to time and location. Nevertheless, very few actual studies of nitrite seasonality in DWDSs exist. Wilczak et al. [4] studied the seasonality of nitrite concentrations in summer and winter conditions and observed that the majority of the water samples with increased nitrite in the DWDS were collected in the summer. However, elevated nitrite concentrations occurred during the winter conditions also. Schullehner et al. [6] conducted a large and systematic research of nitrite in Danish distributed groundwater that contained nitrite pollution, but did not find any seasonality. The number of ammonia oxidizing bacteria (AOB) in DWDSs has been found to be higher in summer than in winter [9,10,11]. In a 15-month study, Pinto et al. [12] found distinct seasonality in the bacterial dynamics of a DWDS using monochloramine. Stanish et al. [13] noted that in DWDSs with seasonal monochloramine use, the monochloramine increased the abundances of nitrifiers and *Nitrospira*-like organisms. In connection to nitrification episodes in DWDSs, *N*-nitrosodimethylamine (NDMA) and other DBPs have also been found to increase [14]. Woods et al. found NDMA to occur in higher concentrations at a lower temperature in a large urban DWDS [15]. 

In Finland, the main source of drinking water is tap water. The consumption of bottled water in 2016 was 14 l cap^−1^, which was only 13% of the European average [16]. Thus, the nitrite formed from the added monochloramine in the DWDSs is ingested with the drinking water and increases the nitrite exposure. Consequently, the production of drinking water is compelled to keep a balance between preserving the good microbiological quality of the distributed water and the nitrite formation in the DWDS. For this purpose, precise knowledge of the nitrite formation is required. In this research, we studied the nitrite concentrations of the distributed drinking water in the Helsinki Metropolitan Area, Finland. The aim of the study was to evaluate how the nitrite exposure originating from the added chloramine is distributed seasonally and what the main drivers leading to seasonality are.

## 2. Materials and Methods

### 2.1. Water Quality Monitoring Data

In the Helsinki Metropolitan Area, the monitoring of the quality of distributed drinking water is organized by the local water company, the Helsinki Region Environmental Services Authority (HSY). The company has two main drinking water treatment plants (WTPs), located at Pitkäkoski and Vanhakaupunki. The annual drinking water production of HSY was 89.7 Mm^3^ in 2015 [17]. Monochloramine is used in both WTPs for secondary disinfection, and nitrite concentrations have been observed to increase in the DWDSs [18,19]. The target ratio of chlorine and ammonia when feeding the chemicals is 4 mg Cl_2_ l^−1^ per 1 mgN l^−1^, and the target concentration of total chlorine in the freshly produced potable water is 0.35–0.4 mg Cl_2_ l^−1^. The general quality of the produced drinking water is displayed in Table 1.

**Figure 1:**
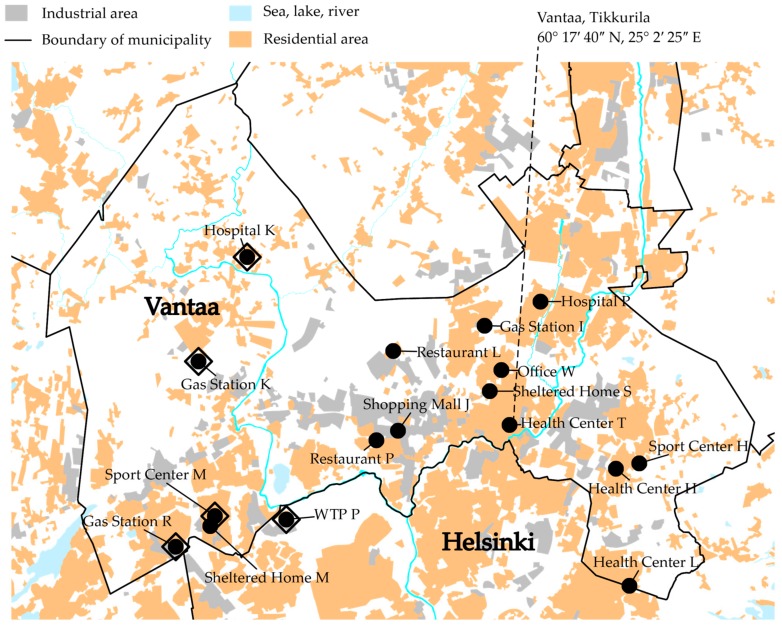
A map of the study area. The black circles are obligatory monitoring locations and the large open diamonds are sampling campaign locations.

The obligatory monitoring of the quality of the distributed drinking water was organized by HSY, and the sampling and analysis were conducted by an accredited laboratory, Metropolilab [21]. In total, the obligatory monitoring data for the cities of Helsinki and Vantaa for the years 2010–2013 included water samples from approximately 900 drinking water taps. However, the data only included 16 locations with a long time series of water quality (lengths: 12–47 months). All these sampling sites were located in the city of Vantaa, inside the DWDS of Pitkäkoski WTP (Figure 1). Nitrite was analyzed from most of the drinking water samples. Thus, the formed nitrite concentration time series consisted of 10–35 analysis results each.

### 2.2. Sampling Campaign

To gather more information on nitrite and other nitrogen compounds in distributed drinking water, a one-year sampling campaign was organized in a section of a DWDS between 1 July 2014 and 2 June 2015 (Figure 1). The section is isolated from the rest of the DWDS in the east, south and north. The Pitkäkoski WTP is situated in the southeastern corner of the section. In the west there are two connections to the rest of the DWDS, one of which is closed. 

We chose four sampling locations that were included in the obligatory monitoring (Sport Center M, Gas Station R, Gas Station K and Hospital K; Figure 1). The drinking water at the Pitkäkoski WTP was sampled from a pipe leading to the laboratory, located at the site of the WTP. Additionally, water at the end of Process Line 1 in the WTP was sampled between 9 December 2014 and 2 June 2015. Sampling tours were organized approximately once a month, with 3–7-week intervals, on Tuesdays starting at 08:00 and ending at 13:00. All the samples were stored in cool boxes with freezer blocks during the sampling tour. The temperatures inside the cool boxes were between 4 °C and 15 °C, depending on the initial temperature of the samples. The samples were analyzed the same day they were collected. If the analysis took more than one day, the samples were stored at a temperature of 4 °C, without preservation.

The sampling procedure included discharging water from the tap before taking the samples. At first, the samples were taken after a 5-min discharge of water from the tap. The water quality change during the discharge was evaluated on 9 September 2014. The results indicated that a discharge of 150–200 l was sufficient to stabilize the water quality according to the ammonium and total chlorine analyses. Thus, the sampling procedure was to discharge at least for 15 min or 200 l of water, depending on the flow of the tap. This sampling procedure was adopted into use from 30 September 2014 to 2 June 2015. Roeselers et al. [22] have also used 15 min extra flushing when sampling drinking water in their study.

The drinking water samples for nitrite, oxidized nitrogen (nitrite + nitrate) and total nitrogen analyses were collected in amber glass bottles with ground-glass stoppers. The samples above were analyzed in the laboratory of the Water and Environmental Engineering Group of Aalto University with standard methods (see Section S1 in the Supplementary Materials). Ammonium concentrations were analyzed in the laboratory of HSY, at the Pitkäkoski WTP. These samples were collected in plastic bottles that were completely filled. The samples were analyzed with the standard method (see Section S1 in the Supplementary Materials). All samples were analyzed as duplicates, and the mean values of the analyses were reported as the analysis results. The ammonium analysis included ammonia, ammonium and chloramines. The limits of quantification (LoQs) of the ammonium and nitrite analyses are provided in the Section S1 in the Supplementary Materials.

### 2.3. Data Collection, Statistical Methods and Calculations

The statistical analyses were executed in MathWorks Matlab and IBM SPSS Statistics. Seasonal indices (*SI*; see Equation (1)) were utilized to inspect the seasonal properties of nitrite time series. The nitrite concentrations were calculated as two-month or three-month averages, because there were gaps in the monthly data. It was not possible to fill all the gaps with this method and the remaining gaps of one concentration were filled with the averages of adjacent two-month concentrations. The gaps of two or more concentrations were not filled. The seasonal indices were calculated from the two-month and three-month averages of nitrite concentrations according to Equation (1):

(1) $$\;SI=\frac{x_{i}}{\hat{x}}\;$$

where SI = seasonal index, based on one-year data, $x_{i}\;$= the mean nitrite concentration in a two-month season i, $\hat{x}\;$= the annual mean nitrite concentration, and i= the numbers of the two-month (*i* = 1–6) or three-month (*i* = 1–4) periods in a year.

The data was depicted as time series and boxplots. The nitrite data in the obligatory monitoring and the sampling campaign were not normally distributed (as assessed by the Anderson-Darling test), and thus we used non-parametric tests. Spearman’s rank-order correlation coefficients were used to define the correlation between two variables, with the limit of significance being *p* < 0.05. The water age was a maximum water age (Table 2), estimated with a model of the DWDS [19,23]. The calculations with the analysis results lower than the LoQ were done with the numerical value of the half of the LoQ. 

## 3. Results and Discussion

### 3.1. Obligatory Monitoring Data

#### 3.1.1. Seasonality of the Nitrite Time Series

**Figure 2:**
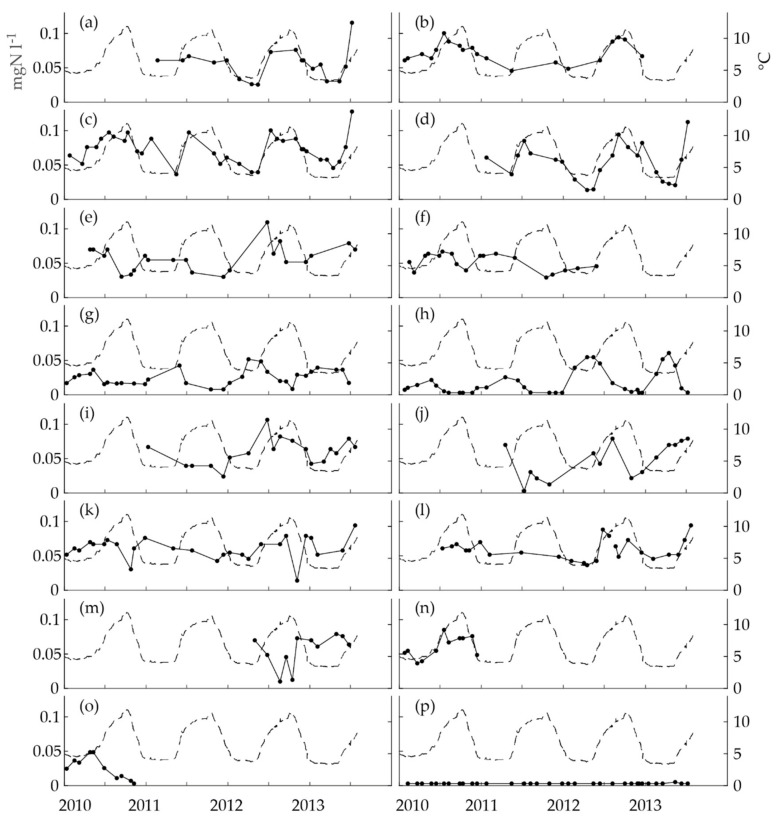
The time series of nitrite concentrations in the drinking water distribution system (DWDS) in the obligatory monitoring data collected between January 2010 and July 2013 (the unbroken line with dots, the left *y*-axis: one dot represents one analysis) and temperature at the WTP (the dashed line, the right *y*-axis) for (**a**) Sheltered Home M, (**b**) Sport Center M, (**c**) Gas Station R, (**d**) Shopping Mall J, (**e**) Gas Station I, (**f**) Health Center H, (**g**) Health Center L, (**h**) Gas Station K, (**i**) Sheltered Home S, (**j**) Restaurant L, (**k**) Hospital P, (**l**) Health Center T, (**m**) Sport Center H, (**n**) Restaurant P, (**o**) Office W and (**p**) Hospital K. The number of nitrite analyses for each time series = 10–35.

The seasonality of nitrite concentrations in the distributed drinking water was first studied in the obligatory monitoring data. Quite a few of the nitrite time series showed distinct seasonality with annual crests and troughs (Figure 2). Nitrification is strongly dependent on temperature; thus, the time series were first inspected visually by comparing them to the time series of the water temperature at the WTP. According to this inspection, the data included time series with a seasonality similar to the seasonality of the temperature (Figure 2a–d), a seasonality reversed to the temperature (Figure 2e–h), time series with variation, but no clear seasonality (Figure 2i–l), short time series (Figure 2m–o) and a time series with no variation (Figure 2p). The first group had crests during the warm period of the year and the second group during the cold period. 

#### 3.1.2. Seasonal Index of a Nitrite Time Series

**Figure 3:**
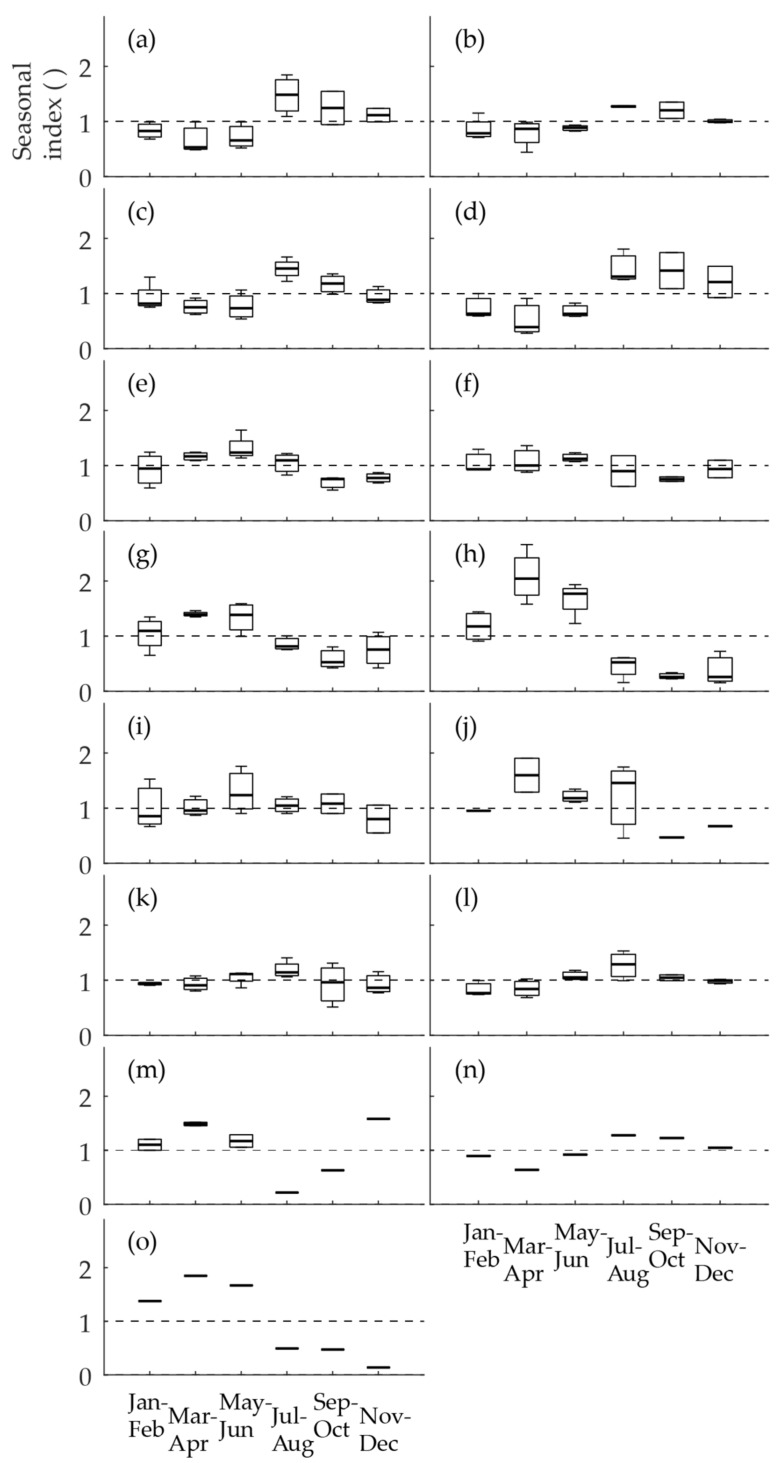
The two-month seasonal indices of the nitrite time series recorded in obligatory monitoring data between January 2010 and July 2013 for (**a**) Sheltered Home M, (**b**) Sport Center M, (**c**) Gas Station R, (**d**) Shopping Mall J, (**e**) Gas Station I, (**f**) Health Center H, (**g**) Health Center L, (**h**) Gas Station K, (**i**) Sheltered Home S, (**j**) Restaurant L, (**k**) Hospital P, (**l**) Health Center T, (**m**) Sport Center H, (**n**) Restaurant P and (**o**) Office W. In the boxplots, the middle values are medians, the boxes are the upper and lower quartiles, and are the whiskers the minima and the maxima.

To conduct a more systematic inspection, we calculated the two-month seasonal indices (Equation (1)) for the single time series. The seasonal indices included 1–3 nitrite values each. We have drawn boxplots of the single-year seasonal indices to depict the inter-year variation of the values. In nine of the time series, the seasonal variation was largely substantial (Figure 3a,c,d,g,h,j,m–o), while in four it was clear but less eminent (Figure 3b,e,f,l). The seasonal index revealed that no clear seasonality existed in two time series, even though there was variation in the values (Figure 3i,k). No seasonal index was calculated for the time series of Hospital K (Figure 2p), because the values did not vary. The evaluation of the seasonal patterns of the time series was decided according to both the time series and the seasonal indices. In equivocal cases, the seasonal index was given more weight.

The time series were found to be of four types. Firstly, the annual maximum occurred during the warm period of the year (months 7–12) and the minimum during the cold period (months 1–6). This type of series was called a summer maximum and included the time series of Sheltered Home M, Sport Center M, Gas Station R, Shopping Mall J, Health Center T, and Restaurant P. Secondly, the nitrite maximum concentrations occurred during the cold period and the minimum concentrations occurred during the warm period. This type was called a winter maximum and included the time series of Gas Station I, Health Center H, Health Center L, Gas Station K, Restaurant L, Sport Center H and Office W. The third type indicated no clear seasonality, though the concentrations varied over time. This type was called mixed seasonality and included the time series of Sheltered Home S and Hospital P. Finally, one time series, that of Hospital K, with no variation and values below the LoQ, formed the fourth type, was called no seasonality. 

We included the short one-year time series to the relevant seasonality groups, because the seasonality of distributed drinking water quality is quite often studied in time series with only one year of data [4,15,24]. Additionally, the seasonal patterns in the time series (Figure 2m–o) and in the seasonal indices (Figure 3m–o) were quite clear in all three cases. 

#### 3.1.3. Nitrification in the Sampling Line Running to the on-Site Laboratory

**Figure 4:**
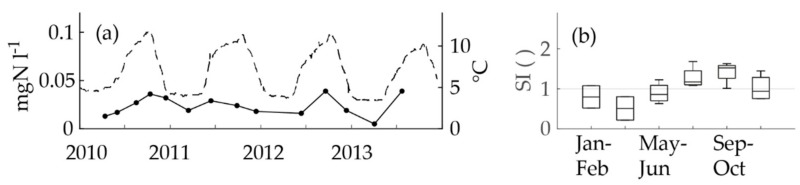
The parts of the figure: (**a**) The nitrite concentrations time series (black dots and an unbroken line; the left *y*-axis; one dot represents one analysis) and the temperature at the WTP (the dashed line; the right *y*-axis) and (**b**) a boxplot of the seasonal indices of the nitrite (each seasonal index included 1–2 nitrite analyses) in the distributed water from the laboratory tap at the Pitkäkoski WTP in the in-house control data recorded between 2010 and 2013. In the boxplot, the middle line is the median, the boxes are the lower and upper quartiles and the whiskers are the minima and maxima.

**Figure 5:**
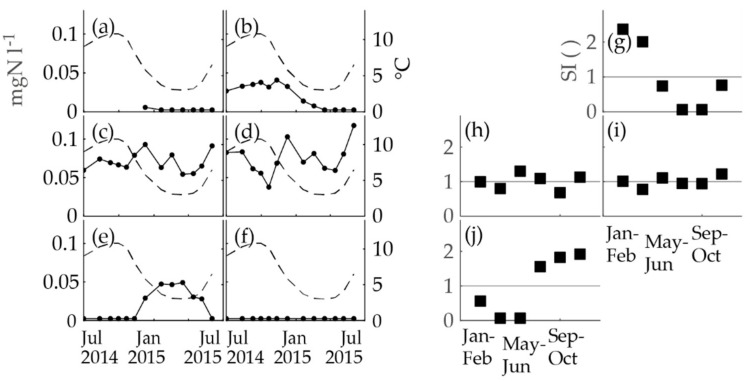
The nitrite concentrations (the black dots and an unbroken line; the left *y*-axis) compared to the temperature (the dashed line; the right *y*-axis) at the WTP and the two-month seasonal indices of nitrite concentrations (the black squares, *N* of nitrite analyses for each box = 2–3) of the sampling campaign. The parts of the figure are as follows: (**a**) nitrite at Pitkäkoski WTP (Process Line 1), (**b**) nitrite at Pitkäkoski WTP (a tap in the on-site laboratory), (**c**) nitrite at Sport Center M, (**d**) nitrite at Gas Station R, (**e**) nitrite at Gas Station K, (**f**) nitrite at Hospital K, (**g**) seasonal indices of nitrite at Pitkäkoski WTP (a tap in the on-site laboratory), (**h**) seasonal indices of nitrite at Sport Center M, (**i**) seasonal indices of nitrite at Gas Station R, and (**j**) seasonal indices of nitrite at Gas Station K.

Unexpectedly, the nitrite concentrations at the Pitkäkoski WTP followed a seasonal pattern (Figure 4a). The pattern had a crest in the warm period and a trough in the cold period. The nitrite concentrations varied between 0.005 mgN l^−1^ and 0.039 mgN l^−1^ (median 0.019 mgN l^−1^). The seasonal indices confirmed the seasonality (Figure 4b). However, nitrite could not have been formed in the treatment process, because monochloramine was added to the drinking water as the last step. According to the theoretical calculations by Fleming et al. [25] the monochloramine dose (Table 1) was not high enough to prevent nitrite formation in the biofilm [19]. The tap from which samples were taken had a constant uninterrupted flow of 1.9 l min^−1^. The retention time of the sampling line is not known, but most probably it amounts to minutes or tens of minutes at maximum. Supposedly, chloramine was decomposed and ammonium was nitrified into nitrite in the biofilm formed inside the sampling line. This supposition was confirmed during the sampling campaign. However, the total microbes (Table 1) were below 10 cfu ml^−1^, with one exception of 45 cfu ml^−1^, which indicated that the formation of the possible biofilm was not extensive. In the middle of the sampling campaign, the WTP personnel noticed that the nitrite and ammonium concentrations were different at the end of the process line compared to the sampling tap. The maximum concentration after the final step of the process line was 0.0057 mgN l^−1^ and at the tap in the laboratory it was seven-fold: 0.041 mgN l^−1^ (Figure 5a,b). Thus, most probably the nitrite concentrations in the water entering the DWDS were close to or below the LoQ. 

### 3.2. Sampling Campaign

The nitrite concentrations at the locations of Pitkäkoski WTP, Sport Center M, Gas Station R, Gas Station K and Hospital K, were analyzed in a separate sampling campaign to complement the findings based on the obligatory monitoring data. The same methods as above were utilized to inspect the seasonality of the obligatory monitoring data (Figure 5). The nitrite concentration time series were compared to the temperature at the Pitkäkoski WTP (Figure 5a–f). Seasonality occurred in two time series: the taps at Pitkäkoski laboratory and Gas Station K. On the contrary, the time series of Sport Center M and Gas Station R had variation, but no clear seasonality. The seasonal indices confirmed these observations (Figure 5g–j). The time series of Hospital K was flat with no variation (Figure 5f). As a summary, three out of five time series behaved similarly seasonally as in the obligatory monitoring and in-house control data of the WTP (WTP P, Gas Station K, and Hospital K) and two behaved differently (Sport Center M and Gas Station R). 

At Sport Center M and Gas Station R, seasonality was not observed in the data of the sampling campaign, which, however, did not necessarily mean that there was no seasonal behavior of nitrite concentrations. The longer time series of obligatory monitoring had already proved that seasonality featured in the nitrite concentrations of these sampling locations, and the one-year data of the sampling campaign did not override this notion. Furthermore, the median values of the nitrite concentrations of Sport Center M and Gas Station R in the sampling campaign and obligatory monitoring were almost equal (Sport Center M: 0.067 mgN l^−1^ and 0.068 mgN l^−1^; Gas Station R: 0.070 mgN l^−1^ and 0.072 mgN l^−1^ respectively) and the ranges overlapped (Sport Center M: 0.054–0.093 mgN l^−1^ and 0.028–0.10 mgN l^−1^; Gas Station R: 0.038–0.118 mgN l^−1^ and 0.037–0.128 mgN l^−1^ respectively). The explanation for the mixed seasonal behavior in these locations is not known; however, a possible explanation is variations in the water consumption in the premises.

### 3.3. Evaluation of the Methods

The seasonal index is routinely used in detecting the seasonal patterns of time series, especially in economic analysis [26,27]. Biochemical reactions in DWDSs follow intra-year changes, pre-eminently of temperature, and thus it is reasonable to study nitrite formation with methods designed to understand seasonal properties. A seasonal index is a simple method to evaluate seasonality. More advanced methods, for example an ARIMA time series analysis, could not be used, because the sampling intervals were not equidistant. 

Visual inspection was an even more simple method than the seasonal index. In addition, the visual categorization of some of the nitrite time series was debatable (e.g., Sheltered Home S in Figure 2i and Restaurant L in Figure 2j). However, the seasonal index helped in this categorization problem. 

To test our method of determining the seasonal index, we calculated another set of seasonal indices of the nitrite time series in the obligatory monitoring data with a different division of the seasons, starting from December and not January. This division of seasons would have fitted better with the seasonality pattern of the temperature, and we wanted to observe whether this division would have altered the seasonality types of nitrite. Most of the time series were classified similarly, compared to the method of starting the division from January (Figure 3). Nevertheless, the method of starting the division from December did not improve the classification of the nitrite seasonality types. On the contrary, the location of Gas Station I was classified as a mixed seasonality, even though it expressed an evident winter maximum according to the nitrite time series (Figure 2e). Nevertheless, this did not affect the final decisions of the seasonality types of the nitrite time series. In addition, we tested a method of three-month division starting from January, which flattened the seasonal features slightly, though it gave a similar classification as the method of the two-month averages starting from January (Figure 3). These additional analyses of the method are provided in Supplementary Materials Section S2.

During the calculation of the seasonal indices, we experimented with filling the gaps in the data. It was found that all the choices of not filling the gaps, filling only one-season gaps with interpolation, or filling all the gaps gave the same final results for the seasonality types. Thus, without additional restrictions, we chose a conservative filling strategy: only one-season gaps were interpolated.

The nitrite time series of Hospital P (Figure 2k), in the obligatory monitoring data, was scrutinized more closely to decide whether it showed seasonality or not. The time series had two sharp peaks, which were considered as possible outliers. When these were removed, the seasonal indices of the six seasons had less variation. It was concluded that nitrite concentrations analyzed from the water at Hospital P did not have annual seasonality according to the available data. Nitrification in the premises’ plumbing system [28] can increase the nitrite concentrations in drinking water. Obviously, the sharp changes downwards in nitrite concentration in Figure 2k were not a result of this phenomenon. It was noted that the low concentrations were both observed on Mondays. We suspected that water may have been stagnating during the weekend, thus possibly causing lower nitrite concentrations. However, it was discovered that most of the samples were taken on Mondays (84% of all samples). Thus, Monday sampling did not implicitly cause decreased nitrite concentrations, but the high percentage of Monday sampling may have caused some other, unknown bias to the nitrite data. 

### 3.4. Nitrite Concentrations in Seasonality Groups

**Figure 6:**
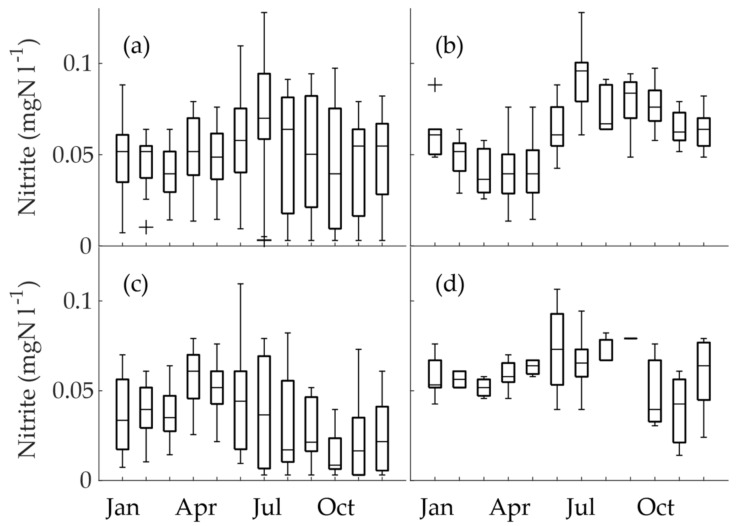
The monthly boxplots of nitrite seasonality in obligatory monitoring data for (**a**) all of the time series (excluding Hospital K, *N* = 317), (**b**) the time series with the summer maximum (*N* = 129), (**c**) the time series with the winter maximum (*N* = 143), and (**d**) the time series with mixed seasonality (*N* = 45).

When all the nitrite concentrations of obligatory monitoring were combined (excluding monitoring for Hospital K) and depicted as a monthly boxplot (Figure 6a), seasonality was only observable to a lesser extent. The highest monthly median and maximum nitrite concentrations both occurred in July. On the other hand, distinct seasonality can be observed in the nitrite concentrations of the conjoined data of summer maxima (Figure 6b) and winter maxima (Figure 6c). In the summer maximum group (Figure 6b), the highest median nitrite concentrations occurred during July. This is interesting, because it coincided with the sharp rise in temperature, but not the highest temperature. We investigated whether the sampling schedule caused bias and possible higher concentrations, but this was not the case; all the locations included in the group were sampled during July. The winter maximum group was more heterogeneous than the summer maximum group (Figure 6c). The highest monthly median occurred in April. Nevertheless, the highest maximum occurred during June, which again coincided with the sharply rising temperature. In all the months from July to December, the lowest values were below the LoQ. The mixed seasonality group revealed the maximum nitrite in June, but interestingly lacked the lowest nitrite concentrations (below or close to the LoQ; Figure 6d). This was possibly because the low concentrations were masked by the high concentrations, when waters were mixed in the junctions of the DWDS. 

In the Helsinki Metropolitan Area, nitrite levels had been observed to increase in the DWDS in succession to the commissioning of a granular activated carbon (GAC) filtration unit at the Vanhakaupunki WTP to reduce organic matter in the drinking water [18,29]. A similar process is currently in use at the Pitkäkoski WTP. Nevertheless, the nitrite concentrations were somewhat lower in the Vanhakaupunki DWDS (0.005–0.05 mgN l^−1^) compared to our study. 

In a recent study, Schullehner et al. [6] examined nitrite and other nitrogen species at WTPs and DWDSs in a research that included the whole of Denmark. However, they did not find seasonal variation in nitrite concentrations. Their method of evaluating seasonality in their vast data resembled Figure 6a in our research. As we have seen in our research, when large data is inspected as a whole, opposing seasonalities can override each other. These, among other factors, may have hidden the seasonality of nitrite in the Danish study. However, we have to note that the drinking water in Denmark is abstracted from groundwater, which is polluted by nitrite and nitrate. Thus, the nitrite did not originate from monochloramine addition.

### 3.5. Nitrite Seasonality in Relation to Temperature and Water Age 

We studied the relation of the temperature at the WTP and the nitrite concentrations in the obligatory monitoring data with a correlation analysis. The Spearman rank-ordered correlation coefficient of temperature and nitrite in the summer maximum group was 0.69 (*p* < 0.001), in the winter maximum group it was −0.50 (*p* < 0.001), and in the whole nitrite time series data it was merely 0.07 (*p* = 0.23). Thus, the winter maximum group had ostensibly reversed temperature dependence and the whole data did not have any dependence. The kinetic parameters of nitrification depend on temperature following the Arrhenius equation [30], and the maximum growth rate of nitrification bacteria roughly doubles between 12 and 26 °C [31]. Moreover, the number of AOB has been found to be 100–1000 times higher in DWDSs in the summer compared to the winter [9,10,11]. Thus, the reversed correlation requires an explanation.

When the seasonality types of nitrite concentrations were inspected against water age, it was found that in the summer maximum group the water age was shorter (median: 6.7 h; range: 5–64 h) than in the winter maximum group (median: 31 h; range: 27–54 h). In the summer maximum group, the nitrite crests were observed at the median temperature of 9.1 °C (range: 7.7–9.6 °C), and in the winter maximum group they were observed at the median temperature of 5.0 °C (3.7–7.7 °C). Thus, at a low temperature, a longer reaction time for nitrite formation was required, and the contradictory observation of maximum nitrite concentrations in cold temperature resulted from the decelerated ammonium oxidation. 

Possibly, in complicated DWDSs, nitrite seasonality can be distorted by mixing the waters from different directions. As we noticed, water age impacts the nitrite concentrations, and if in a junction of DWDS two or more waters with different water ages and nitrite concentrations were mixed, the result was probably no clear seasonality, or alternating seasonalities, depending on the changing shares of flows from the different directions. This could explain the time series with mixed seasonality.

We observed that there were two types of low nitrite concentrations. Firstly, at low water age, low nitrite concentrations occurred because nitrite had not yet been formed. However, we have to add that nitrite started to form very rapidly when the conditions were favorable, i.e., when the water was in contact with a nitrifying biofilm. As we observed in the sampling line at the WTP, this happened in minutes. Secondly, low nitrite concentrations occurred when nitrite had been oxidized into nitrate. The time series without variation (Hospital K, with a water age of 190 h) was a result of nitrite oxidation occurring completely, with the final nitrogen compound being fully oxidized nitrate. Generally, nitrite occurs in stagnating water, meaning at a high water age [32], which disagrees with our observations. The general observation is probably a result of a higher monochloramine dose (1–3 mgCl_2_ l^−1^ [4]), which prevents nitrification initially, but further in the DWDS the lowered concentration is not capable of inhibiting the AOB inside the biofilm. Our results were received from a DWDS with a low initial monochloramine dose (median: 0.39 mgCl_2_ l^−1^).

Wilczak et al. [4] noted that a higher share of high nitrite occurred during the summer season, but nitrite occurred also during the winter season. This corroborates our findings that nitrification is not totally inhibited during cold temperature, but is slowed down significantly. Moreover, AOB and NOB have been observed in DWDSs elsewhere in Finland, in spite of the low temperatures of water [33]. Unfortunately, this study did not include an evaluation of the seasonality. 

Woods et al. [15] studied the seasonality of NDMA in DWDSs with monochloramine disinfection and utilized the temperature of water as a reference of seasonal behavior of the NDMA concentrations. They noted that the NDMA concentrations increased in temperatures lower than 10 °C. Their approach to evaluating seasonality was somewhat similar to our first method (Figure 2) and strengthens the notion that temperature is a very important background variable in the seasonality of water constituents in DWDSs.

### 3.6. Nitrite’s Correlation to Other Nitrogen Forms in Relation to Seasonality

Generally, when nitrite concentrations increase in the DWDS, ammonium concentrations should decrease accordingly [4]. However, nitrite concentrations of the sampling campaign data (excluding Hospital K) did not correlate negatively or even significantly with ammonium concentrations (Spearman’s rho = 0.04, *p* = 0.79). Nevertheless, ammonium correlated negatively with oxidized nitrogen (nitrite + nitrate) (Spearman’s rho = −0.94, *p* = 1.3 × 10^−24^). Usually, both nitrite and nitrate concentrations increase together in the DWDSs [4,6], but in our data nitrate correlated negatively with nitrite (Spearman’s rho = −0.27, *p* = 0.053). 

**Figure 7:**
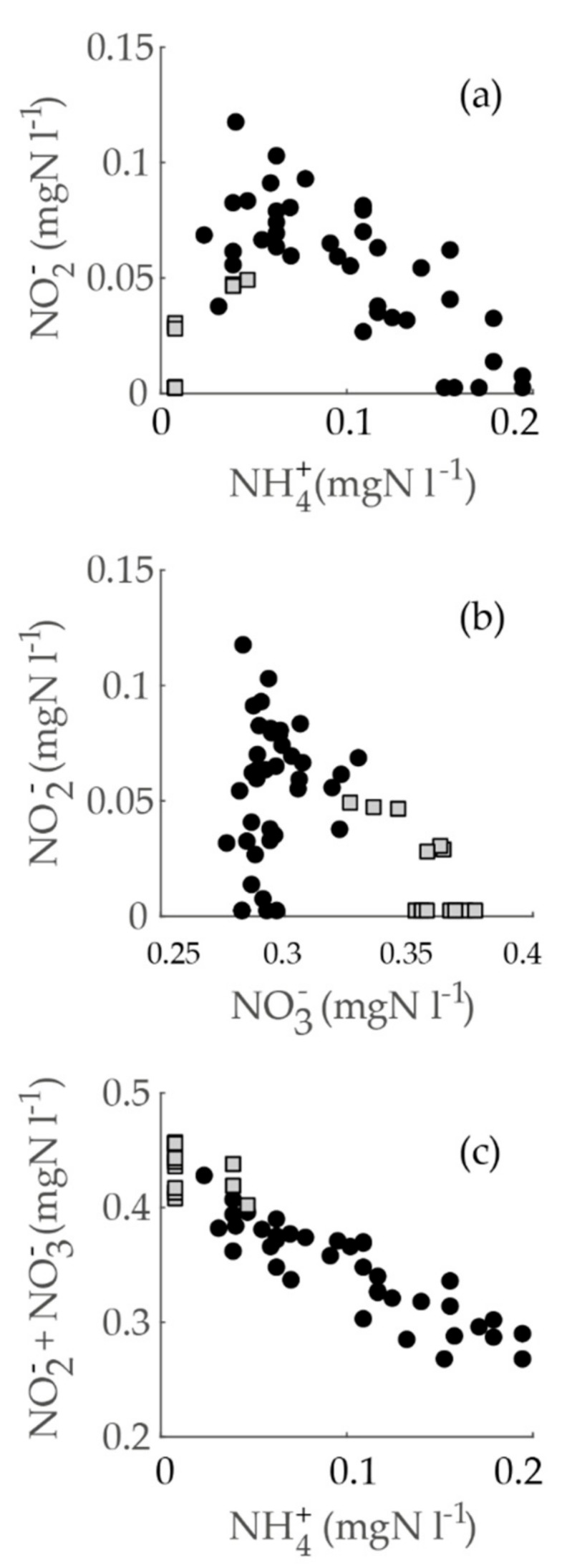
A scatterplot of inorganic nitrogen species in the sampling campaign. Groups are divided by the seasonality type of the sampling location; black dots indicate a summer maximum (in the locations of Pitkäkoski WTP, Sport Center M, and Gas Station R); grey squares indicate a winter maximum (Gas Station K). The graphs show (**a**) ammonium vs. nitrite, (**b**) nitrate vs. nitrite, and (**c**) ammonium vs. nitrite + nitrate.

Next, we inspected the correlation separately in the locations with a summer maximum (Pitkäkoski WTP, Sport Center M, and Gas Station R; water ages up to 6.7 h) and the location with a winter maximum (Gas Station K, water age 33 h) in the sampling campaign data. Furthermore, we noticed that the ammonium and nitrite scatterplot suggested a curvilinear dependence (Figure 7a), which could have resulted from two subgroups with different types of correlations. This appeared to be accurate: the correlation of ammonium and nitrite was clearly negative and significant in the locations with a summer maximum (Spearman’s rho = −0.69, *p* =1.4 × 10^−6^). On the other hand, in the data with a winter maximum, a positive correlation appeared (Spearman’s rho = 0.80, *p* = 0.001). Both of these correlations were more significant than the correlation in the combined data. Furthermore, the correlations indicated that, at low water ages, ammonium decreased while nitrite increased, but at a higher water age, both nitrite and ammonium decreased. The latter interpretation was corroborated by the fact that both nitrite and ammonium were below the LoQ at the farthest point examined (Hospital K) with the water age of 190 h. 

A similar inspection was conducted for the correlations of nitrite and nitrate (summer maximum, Spearman’s rho = 0.25, *p* = 0.12; winter maximum, Spearman’s rho = −0.63, *p* = 0.02). Thus, at lower water ages, nitrite and nitrate both increased, but at a higher water age nitrite decreased while nitrate increased (Figure 7b). Additionally, when we inspected the correlation of ammonium to oxidized nitrogen, the correlations inside subgroups were of the same sign (summer maximum, Spearman’s rho = −0.90, *p* = 2.7 × 10^−15^; winter maximum, Spearman’s rho = −0.43, *p* = 0.15). On a scatterplot, the correlation was evidently linear, and the subgroups did not stand out from the whole of the data (Figure 7c). 

Thus, according to the correlation analysis of the nitrogen species, ammonium oxidation into nitrite was the dominant reaction in the summer maximum group with lower water ages, and nitrite oxidation into nitrate was the dominant reaction in the winter maximum group with a higher water age. 

### 3.7. Denitrification

**Figure 8:**
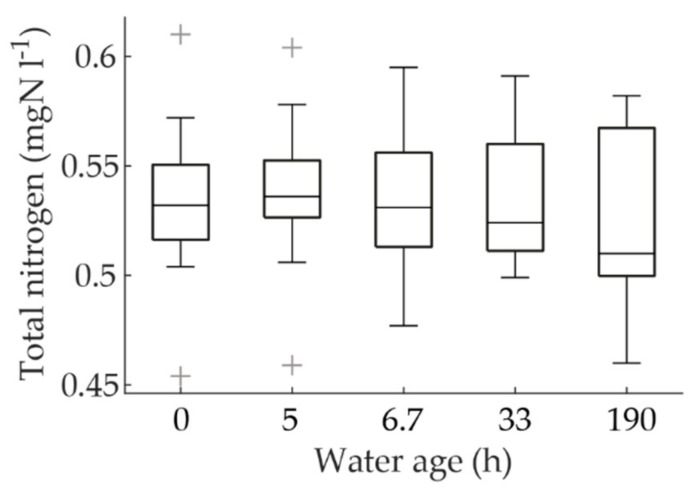
The variation of the total nitrogen concentrations by water age.

Denitrification organisms have been observed in DWDSs [34]. Denitrification can decrease the nitrite and nitrate concentrations if the conditions in the biofilm are anoxic. This could have impacted our interpretation of the decreasing nitrite concentrations. To investigate whether denitrification played a significant role, the total nitrogen data of the sampling campaign at different water ages was plotted as a boxplot (Figure 8). A slight decline in total nitrogen, especially at the water age of 190 h, can be observed. On the other hand, the non-significant Spearman correlation of the data (Spearman’s rho = −0.13, *p* = 0.29) did not corroborate the observation. However, the possibility of denitrification was not completely excluded, even though the extent of denitrification was evidently small. When the sum of nitrogen species (ammonium, nitrite, and nitrate) was investigated, it was noted that the concentrations did not decline with increasing water age and the Spearman correlation with water age was insignificant (Spearman’s rho = 0.08, *p* = 0.53). This implies that the decline in total nitrogen was not inorganic nitrogen, but organic nitrogen, which is included in the analysis of total nitrogen. Furthermore, it is probable that the loss of nitrogen at water ages of 33 and 190 h was due to assimilation into the growth of microorganisms. The loss of total nitrogen at the water age of 190 h was 2.7% from the mean total nitrogen of the rest of the data of the sampling campaign. This percentage is close to those observed in wastewater treatment, for example, in a sequencing batch reactor the assimilation percentage has been recorded to be 3.7% [35] and in a granular sludge process it has been recorded to be less than 5% of all nitrogen in wastewater sludge [36]. 

### 3.8. Nitrite Exposure 

Nitrite in drinking water increased in the DWDS in the Helsinki Metropolitan Area compared to the water at the WTP. According to our results, the dense population consuming drinking water with water ages below 50–60 h received drinking water with elevated nitrite concentrations due to monochloramine addition. This observation applied even to the lowest studied water ages. The nitrite exposure was unevenly distributed during the year, depending on the water age: lower water ages (median: 6.7 h) implied a maximum nitrite exposure during summer and higher water ages (median: 31 h) during winter. Interestingly, the scattered population consuming drinking water with the highest water age was least exposed to nitrite. In a previous study of this region, it was noted that the area of elevated nitrite was slightly larger at a temperature below 9 °C than above 9 °C, suggesting that a larger population was exposed to nitrite at the lower temperature [19]. Our findings of seasonality in winter months, due to delayed nitrite formation, corroborate this observation. 

In another earlier study in the Helsinki Metropolitan Area, the increase of nitrite in the Vanhakaupunki DWDS due to the commissioning of a GAC filtration to remove organic matter, in 1998, was totally unexpected [18,29]. It was speculated that AOB increased in the DWDS because of the lessened competition from heterotrophic microorganisms, though definite reasons were not detected [29]. Recently, HSY has been considering introducing membrane filtration at the WTPs to alter the process of organic matter removal [37]. While it is unknown, whether this will influence nitrification or nitrite concentrations in any way, a proper analysis of the nitrite concentrations in the DWDS and the reasons leading to it is advisable before commissioning any process modifications. The altered process may require various other measures in the treatment process to balance good water quality, including extra disinfection. Our results help to design these measures appropriately, taking into account the nitrite exposure of consumers and minimizing it. Also, our results help to notice possible seasonal changes in the concentrations and to direct the monitoring appropriately, taking into account the unexpectedly high winter concentrations. 

In our study, the nitrite concentrations did not exceed the Finnish statutory limit for nitrite (0.15 mgN l^−1^); the highest concentrations in the obligatory monitoring data were 85% of the limit, and in the sampling campaign data they were 78%. However, the earlier Finnish limit of 0.03 mgN l^−1^ from 1994 [38] was exceeded in 70% of the obligatory monitoring data and in 50% of the sampling campaign data. The lower limit is still use, for example, in Denmark [39]. The median of the obligatory monitoring data was 0.052 mgN l^−1^, which suggests that roughly half of the consumers in the area were exposed to concentrations of this level or higher. To perform a more accurate epidemiological assessment of the population exposed to nitrite, a study combining the spatial population data with the spatial nitrite data is required.

## 4. Conclusions

We studied the seasonal variation of nitrite concentrations in a DWDS with monochloramine disinfection in the Helsinki Metropolitan Area. In Finland, tap water is the main source of drinking water, and thus nitrite in tap water increases the nitrite exposure. We used two methods to inspect the seasonal variation of nitrite concentrations: comparing nitrite time series to the water temperature time series and calculating seasonal indices of the nitrite concentrations. The main drivers of nitrite seasonality were the temperature and the water age. We observed that with low water ages (median: 6.7 h), the highest nitrite exposure occurred during the summer months and with higher water ages (median: 31 h) during the winter months. With the highest water age (190 h), nitrite concentrations were the lowest. The dense population closer to the WTP was exposed to higher nitrite concentrations than the scattered population receiving stagnating drinking water. At a low temperature, the winter maxima were caused by the decelerated ammonium oxidation. The correlation inspection of nitrite, ammonium, and nitrate revealed that the dominant reaction at low water ages was ammonium oxidation into nitrite, and at high water ages, it was nitrite oxidation into nitrate. Denitrification did not occur significantly in the DWDS. 

These results help to understand where and when nitrite is formed in the DWDS and direct monitoring appropriately to gain exact knowledge of nitrite exposure. Also, possible future changes in the treatment process and additional disinfection measures can be designed appropriately to minimize extra nitrite exposure.

## Figures and Tables

**Table 1:** The quality of the drinking water at the Pitkäkoski water treatment plant (WTP) from 2010 to 2015. The analyses are from the analytically extensive monitoring which was organized 4–6 times a year by Helsinki Region Environmental Services Authority (HSY) [20].

Analysis	Units	Median	Minimum	Maximum	*N*
Dissolved oxygen	mgO_2_ L^−1^	15.0	12.2	16.1	24
Total chlorine	mgCl_2_ L^−1^	0.39	0.33	0.48	25
Ammonium	mgN l^−1^	0.13	0.10	0.19	23
Temperature	°C	7.5	3.3	11.8	24
pH		8.5	8.0	8.7	25
Hardness	Mmol L^−1^	0.53	0.46	0.62	23
Turbidity	FTU	0.08	0.06	0.12	25
Conductivity	mS m^−1^	15.1	14.2	16.5	24
Total organic carbon	Mg L^−1^	1.6	1.4	2.0	25
Total microbes (R2A)	cfu mL^−1^	<10	<10	45	19
Trihalomethanes	µg L^−1^	<2	<2	<2	23

**Table 2:** The modeled maximum water ages in the sampling locations [19,23].

Location	Water Age (h)	Location	Water Age (h)
Sheltered Home M	5.0	Sheltered Home S	27.3
Sport Center M	5.0	Restaurant L	26.7
Gas Station R	6.7	Hospital P	28.7
Shopping Mall J	63.6	Health Center T	12.9
Gas Station I	31.2	Sport Center H	47.1
Health Center H	53.9	Restaurant P	5.8
Health Center L	29.7	Office W	30.4
Gas Station K	33.0	Hospital K	190

## Supplementary Materials

The following are available online at http://www.mdpi.com/1660-4601/15/8/1756/s1, Section S1. The Analytical Methods: Table S1; Section S2. Additional Methods for Evaluating the Seasonality of Nitrite Concentrations: Figures S1 and S2.
