# Supplementary material for: The Seasonality of Nitrite Concentrations in a Chloraminated Drinking Water Distribution System
Source: Int J Environ Res Public Health. 2018 Aug 15;15(8):1756. doi: 10.3390/ijerph15081756 (PMC6121643; doi:10.3390/ijerph15081756)
Supplement: Supplementary file 1 [file ijerph-15-01756-s001.pdf]

The Seasonality of Nitrite Concentrations in a Chloraminated Drinking Water Distribution System

Pirjo-Liisa Rantanen, Ilkka Mellin, Minna M. Keinänen-Toivola, Merja Ahonen and Riku Vahala

Supplementary materials

Section S1. The Analytical Methods

Table S1. The analytical methods used in analyzing the drinking water samples.

Analysis	Method	Details	LoQ	
Total nitrogen (N_{tot})	SFS-EN ISO 11905-1, 1998 and Standard Methods 2005. 4500 B. Ultraviolet Spectrofotometric Screening Method [1, 2]	Lange Ganimede N Analyzer		Water and environmental engineering, Aalto University, Espoo
Nitrite nitrogen (NO_2^--N)	SFS-EN ISO 13395, 1997 [3]	Flow injection analysis method with FOSS Tecator, FIAstar 5000 Analyzer and Sampler 5027	0.01 mgN l^{-1} according to the standard	-"-
Nitrite + nitrate nitrogen (NO_2^- -N+ NO_3^- -N)	SFS-EN ISO 13395, 1997 [3]	Flow injection analysis method with FOSS Tecator, FIAstar 5000 Analyzer and Sampler 5027		-"-
Ammonium nitrogen (NH_4^+-N)	ISO 7150/1, 1984 [4]	Hach DR28000 spectrophotometer, Hach ammonium cuvette test LCK304	0.015 mgN l^{-1} according to the test kit's instructions [4]	Pitkäkoski laboratory, HSY, Helsinki

Section 2. Additional Methods for Evaluating the Seasonality of Nitrite Concentrations

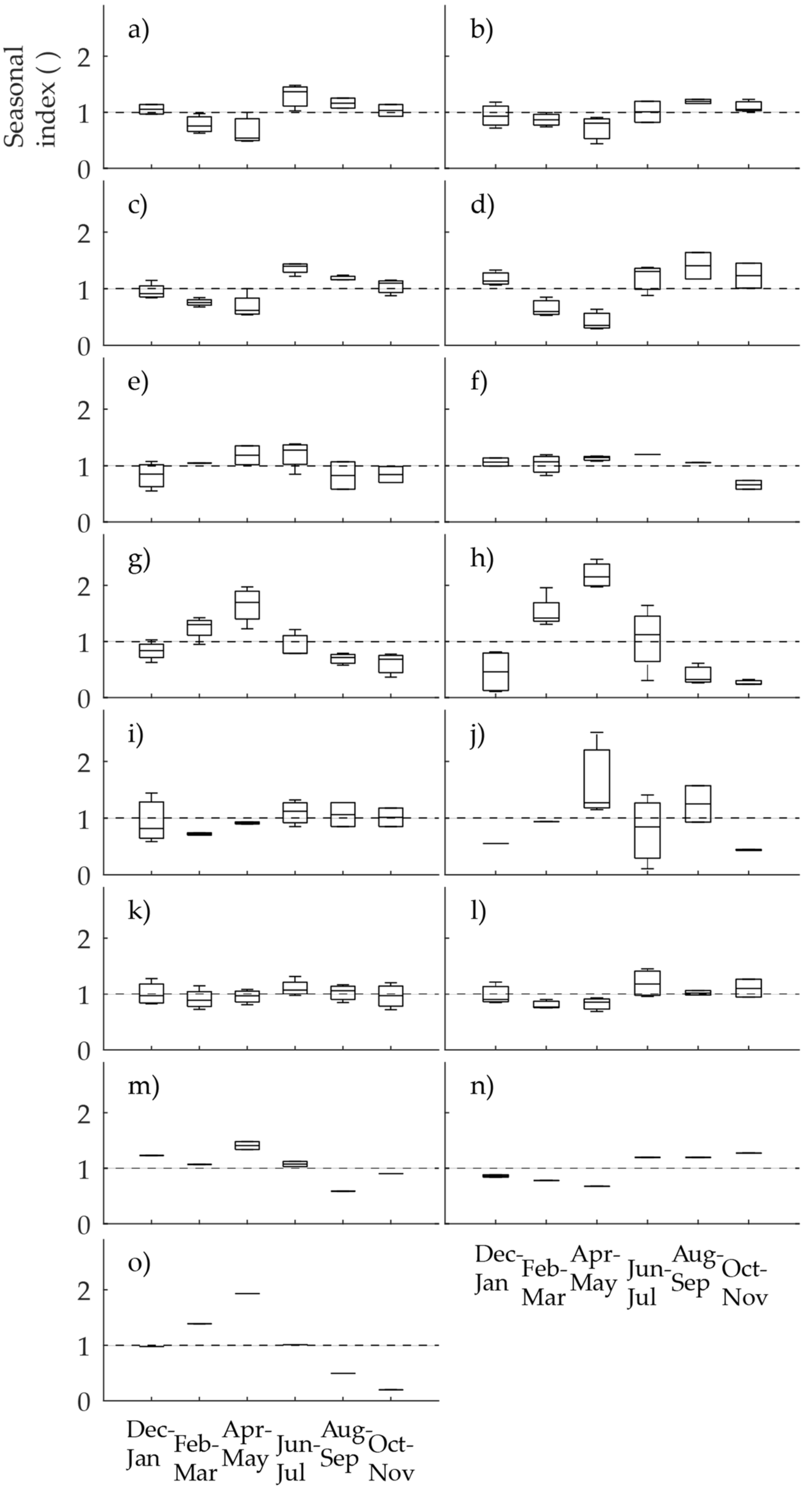

Figure S1. The two-month seasonal indices of the nitrite time series recorded in obligatory monitoring data between January 2010 and July 2013 for (a) Sheltered Home M, (b) Sport Center M, (c) Gas Station R, (d) Shopping Mall J, (e) Gas Station I, (f) Health Center H, (g) Health Center L, (h) Gas Station K, (i) Sheltered Home S, (j) Restaurant L, (k) Hospital P, (l) Health Center T, (m) Sport Center H, (n) Restaurant P and (o) Office W. In the boxplots, the middle values are medians, the boxes are the upper and lower quartiles, and are the whiskers the minima and the maxima. The division of the two-month seasons starts from December.

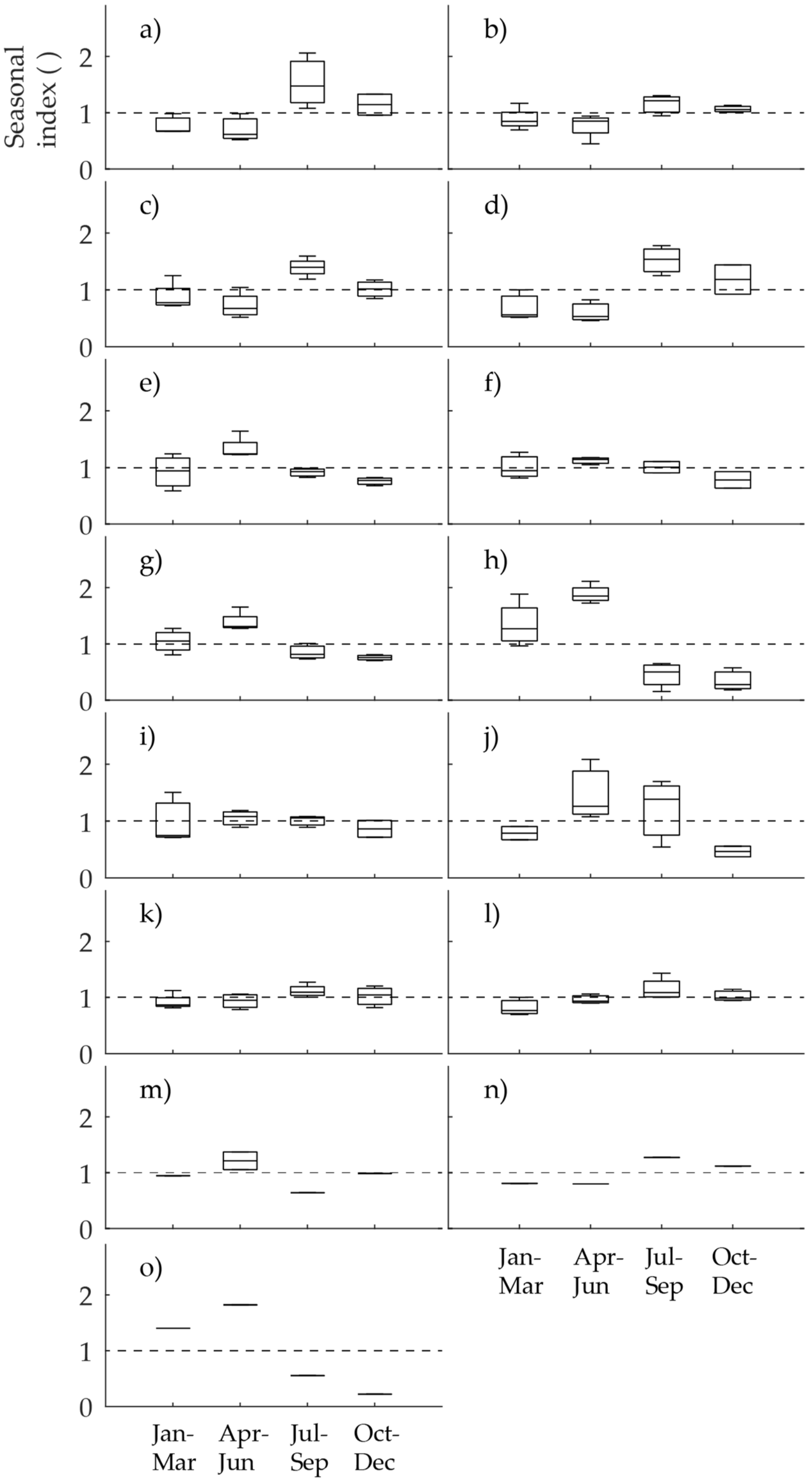

Figure S2. The three-month seasonal indices of the nitrite time series recorded in obligatory monitoring data between January 2010 and July 2013 for (a) Sheltered Home M, (b) Sport Center M, (c) Gas Station R, (d) Shopping Mall J, (e) Gas Station I, (f) Health Center H, (g) Health Center L, (h) Gas Station K, (i) Sheltered Home S, (j) Restaurant L, (k) Hospital P, (l) Health Center T, (m) Sport Center H, (n) Restaurant P and (o) Office W. In the boxplots, the middle values are medians, the boxes are the upper and lower quartiles, and are the whiskers the minima and the maxima. The division of the three-month seasons starts from January.

References

1 Suomen standardisoimisliitto SFS. SFS-EN ISO 11905-1. Water quality. Determination of nitrogen. Part 1: Method using oxidative digestion with peroxodisulfate. 1998, pp. 1+2+13-102013,

2 Franson M.A.H. *Standard Methods for the Examination of Water and Wastewater,* 21st ed.; American Public Health Association; American water Works Association; Water Environment Federation. Washington DC, USA, 2005; ISBN ISBN 0-87553-047-8.

3 Suomen Standardisoimisliitto SFS. SFS-EN ISO 13395. Water quality. Determination of nitrite nitrogen and nitrate nitrogen and the sum of both by flow analysis (CFA and FIA) and spectrometric detection. 1997, 1+27 p.,

4 Suomen standardisoimisliitto SFS. 3032. Determination of ammonia-nitrogen of water. 1976, 6 p.

5 Anon. (2017) Ammonium cuvette test 0.015-2.0 mg/L NH4-N. Hach, UK. https://uk.hach.com.
